# SOCIAL SUPPORT AND HEALTHCARE-SEEKING BEHAVIOR OF OLDER PERSONS IN NIGERIA

**DOI:** 10.1093/geroni/igz038.518

**Published:** 2019-11-08

**Authors:** Funmi Togonu-Bickersteth, Joshua O Aransiola, Catherine O Oyetunji-Alemede, Opeyemi Ekundayo, Oluwasegun Oluwaleimu

**Affiliations:** 1 Obafemi Awolowo University, Ile-Ife, Ile-Ife, Osun State, Nigeria; 2 Obafemi Awolowo University, Ile-Ife, Osun State, Nigeria; 3 OBAFEMI AWOLOWO UNIVERSITY, ILE IFE, OSUN, Nigeria

## Abstract

Abstract The choice of healthcare facility by older persons is an important factor in their healthcare seeking behavior, and this can be associated with a number of factors. This study investigated the relationship between social support and healthcare facility choice of older persons in Nigeria. Other factors associated with the choice of healthcare facilities by older persons were also identified. Quantitative data were collected from a sample of 3,696 elderly aged 60 years above (55.6% male; 44.4% females; mean age = 69.2, SD = 8.60) who were selected through multi-stage systematic random sampling. Binary logistic regression analysis revealed that older persons who received social support were more likely to seek treatment in formal healthcare facilities, while older persons who did not receive any form of social support were more likely to seek treatment in informal healthcare facilities. Membership of social or religious groups was found to be a predictor of health seeking behavior among the older adults. Sex, age, level of education, and ability to handle activities of daily living (ADL), and ease of access to the nearest health facility, were found to be significantly associated with choice of healthcare facilities. The article concludes that there is need for conscious planning to provide formal supports to ease access of older persons to available health facilities. Such facilitation should include financial support and removal of existing physical and cultural barriers to health care utilization by older persons.

